# The impact of non-pharmaceutical interventions on premature births during the COVID-19 pandemic: a nationwide observational study in Korea

**DOI:** 10.3389/fped.2023.1140556

**Published:** 2023-06-27

**Authors:** Ji Young Lee, Joonsik Park, Myeongjee Lee, Minkyung Han, Inkyung Jung, Sung Min Lim, Jee Yeon Baek, Ji-Man Kang, Min Soo Park, Jong Gyun Ahn

**Affiliations:** ^1^ Department of Pediatrics, Severance Children's Hospital, Yonsei University College of Medicine, Seoul, Republic of Korea; ^2^ Division of Neonatology, Department of Pediatrics, Severance Children's Hospital, Yonsei University College of Medicine, Seoul, Republic of Korea; ^3^ Biostatistics Collaboration Unit, Department of Biomedical Systems Informatics, Yonsei University College of Medicine, Seoul, Republic of Korea; ^4^ Division of Biostatistics, Department of Biomedical Systems Informatics, Yonsei University College of Medicine, Seoul, Republic of Korea; ^5^ Institute for Immunology and Immunological Disease, Yonsei University College of Medicine, Seoul, Republic of Korea; ^6^ Pharmaceutical Medicine and Regulatory Science, Yonsei University Graduate School, Seoul, Republic of Korea; ^7^ Department of Clinical Pharmacology, Severance Hospital, Seoul, Republic of Korea

**Keywords:** COVID-19, lockdown, non-pharmaceutical intervention, prematurity, preterm birth

## Abstract

**Background:**

Non-pharmaceutical interventions (NPIs), such as social distancing and hand washing, have been associated with a decline in the preterm birth rate worldwide. We aimed to evaluate whether the preterm birth rate in Korea during the coronavirus disease 2019 lockdown has changed compared to that in previous years.

**Method:**

A birth registry from the Korea Statistical Information Service, which is a nationwide official database, was used to include all births claimed to have occurred between 2011 and 2020. Newborns with gestational age (GA) less than 22 weeks and birth weight less than 220 g were excluded. The pre-NPI period was designated as January 2011 to January 2020, and the NPI period was defined as February 2020 to December 2020. We assessed the effect of NPI on the incidence of prematurity per 100 births using an interrupted time-series quasi-experimental design and implementing an autoregressive integrated moving average (ARIMA) model.

**Results:**

From 2011 to 2020, a total of 3,931,974 live births were registered, among which 11,416 were excluded. Consequently, the final study population included 3,920,558 live births (both singleton and multiple births) among which 275,009 (7.0%) were preterm. The preterm birth rate was significantly higher during the NPI period (8.68%) compared to that in the pre-NPI period (6.92%) (*P *< 0.001). The ARIMA model showed that in all singleton and multiple births, except those in July (observed 9.24, expected 8.54, [95% prediction interval {PI} 8.13–8.96], percent difference 7.81%), September (observed 7.89, expected 8.35, [95% PI 7.93–8.76], percent difference −5.66%), and December (observed 9.90, expected 9.40, [95% PI 8.98–9.82], percent difference 5.2%), most observed values were within the 95% PI of the expected values and showed an increasing trend.

**Conclusion:**

In this nationwide observational study, the trend in premature birth rate did not significantly change due to NPI implementation in Korea, as it had been increasing since 2011. The trend of Korea's birth rate appears to be unaffected by the implementation of NPIs; however, further studies with a longer follow-up period are needed.

## 1. Introduction

The coronavirus disease 2019 (COVID-19) pandemic has brought about unexpected changes in the global society. After the first quarter of 2020, most countries decided to implement non-pharmaceutical interventions (NPIs) to prevent the spread of COVID-19 (1). NPIs consist of frequent hand washing, isolating infected individuals, wearing masks, closing schools and public facilities, and canceling or postponing large gatherings (2). These interventions provided effective measures to control the contagious disease; however, they also led to unwanted and often unexpected public health consequences (3).

Preterm labor is defined as labor that starts before 37 weeks of pregnancy. The estimated global preterm birth rate in 2014 was 10.6%, and this was similar in Asia, where a 10.4% preterm birth rate was reported (4). In recent decades, there has been a trend of increasing preterm birth rates in developed countries (5). Several reports have noted the severe adverse effects of maternal COVID-19 and its associated negative perinatal outcomes for newborns; however, there have also been findings of a potentially decreased rate of preterm births during the pandemic (6).

Investigators in Tennessee first identified an association between NPI and preterm birth after the COVID-19 pandemic (7). Birth records in Tennessee showed a decline in preterm births after the state's stay-at-home order was put into place in 2020. A single center study from South Korea also suggested the possible preventative effects of NPI on preterm birth rates due to the mitigation effects of NPI. Similar phenomena were observed in three Scandinavian countries; however, statistically significant impact of NPI on preterm birth rates was not observed in these studies (8). However, recent studies investigating the effect of NPI on preterm birth rates have demonstrated inconsistent findings. An increased focus on hygiene, strict physical distancing, and home confinement have been suggested as possible reasons for the potential effects of NPI on preterm birth, which may have influenced the overall inflammatory state of pregnant women (9). Therefore, the objective of this study was to establish whether a correlation exists between preterm birth rates and the NPI period in Korea in 2020.

## 2. Materials and methods

### 2.1. Data source

Birth registry data from the Korean Statistical Information Service (an official national database) was used to include information on all births between 2011 and 2020 (10). Parents are required to register a newborn's birth with the Korean government within 1 month of birth, and the data includes not only information about the newborn but also parents’ personal information such as age, location of delivery, and educational level. Among preterm live births, both singleton and multiple births were included for analysis.

### 2.2. Study design

**Figure 1:**
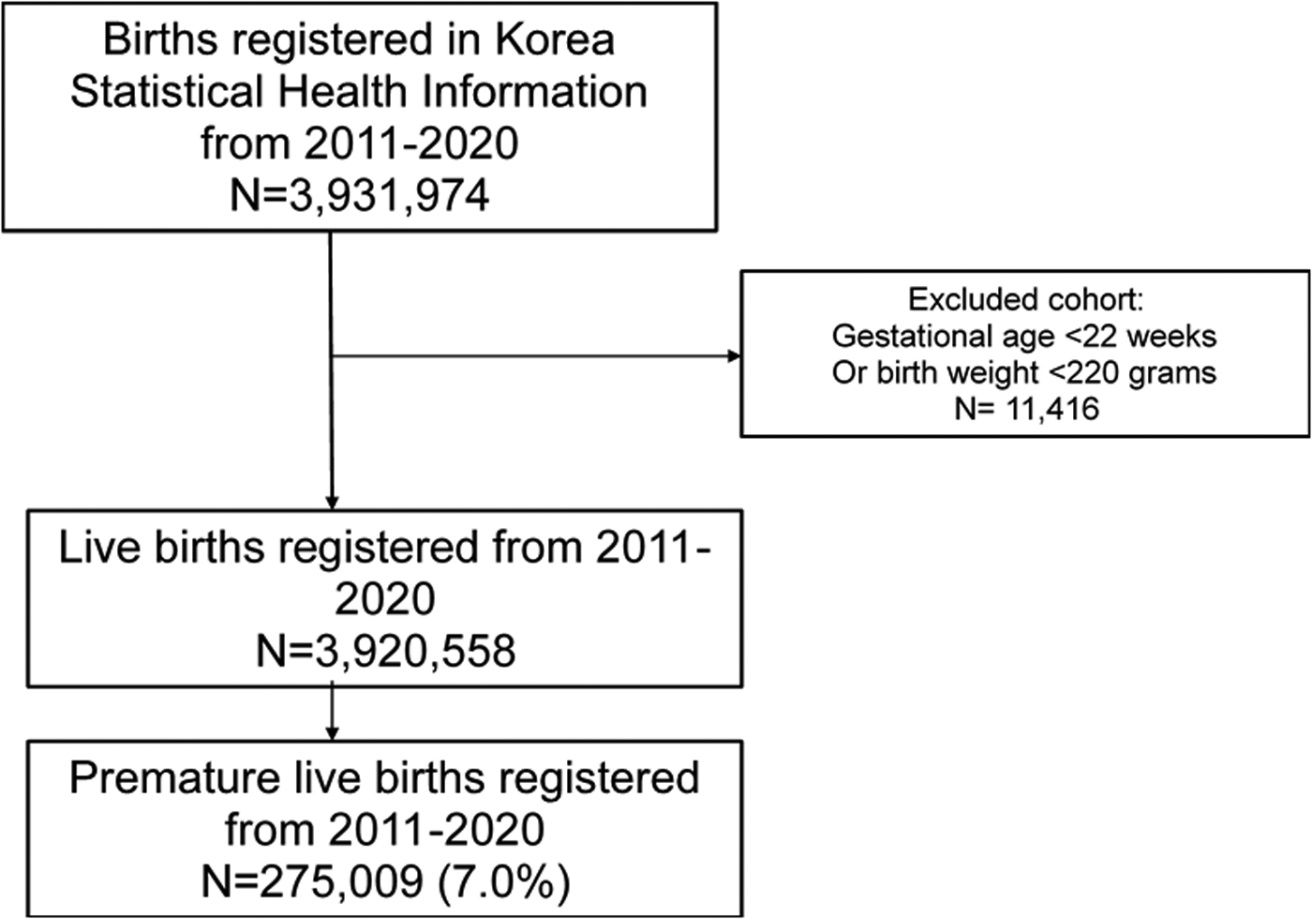
Flow chart of the selection of study population.

This was a retrospective, observational study that evaluated the change in preterm birth rates after the implementation of NPI. After the first confirmed case of COVID-19 in Korea on January 20, 2020, the government imposed mitigation measures, such as social distancing and restricted overseas travel, in February 2020. In this study, the NPI period was defined as January 2011 to January 2020 and the pre-NPI period as February 2020 to December 2020. Although only live births are meant to be registered, we observed 11,416 cases in the categories of gestational age (GA) less than 22 weeks and birth weight less than 220 g (Figure 1). These cases, generally regarded as medically nonviable, were excluded (11, 12).

### 2.3. Definitions

As per the World Health Organization's definition, in this study, preterm birth was defined as births with a GA < 37 weeks (13). Subgroup analysis was conducted by further categorizing the data into the following GA groups: extremely preterm (22 to <28 weeks), very preterm (28 to <32 weeks), and moderate to late preterm (32 to <37 weeks). Small for gestational age (SGA) was defined as a birth weight of less than the 10th percentile for GA and large for gestational age (LGA) as a birth weight of more than the 90th percentile for GA (14).

### 2.4. Statistical analysis

Categorical variables were expressed as frequencies (%) and compared using the Chi-square test. Odds ratios of preterm births by birth types, such as singletons and multiples, and GA subgroups were calculated using multivariable logistic regression analyses between the defined pre-NPI and NPI periods after adjusting for maternal age, sex, maternal education, and birth order.

We assessed the effect of NPI on preterm birth rates and prematurity incidence per 100 births using an interrupted time-series quasi-experimental design (15). To account for seasonality and autocorrelation in preterm rates over time, an autoregressive integrated moving average (ARIMA) model was implemented in the data from the pre-NPI period. The optimal ARIMA parameters were identified based on an automated algorithm, specifically, *auto.arima()* in the *forecast* package in R (16). Using the optimal model selected for each outcome, we derived the expected preterm birth rates after NPI implementation and the percentage difference between the observed and expected preterm birth rates to evaluate the prediction effect. An “unexpected” outcome was defined as an observed value that was outside the expected 95% prediction interval (PI). As a sensitivity analysis, we implemented a standard interrupted time analysis with a segmented regression model allowing level and slope change to evaluate whether the trend in monthly prematurity birth rate incidence changed in the NPI period when compared to the pre-NPI period. A *P-*value < 0.05 was considered statistically significant. Statistical analyses were performed using SAS version 9.4 (SAS Institute Inc., Cary, NC, USA) and R Statistical Software (version 4.1.2; R Core Team 2021).

Additionally, to compare the results using different statistical methods, segmented regression analysis was performed for statistical modeling of interrupted time series to evaluate whether the trend changed during the NPI period (17, 18). The results of this analysis are included in the supplementary material.

### 2.5. Ethics

This research was conducted ethically, in accordance with the World Medical Association and Declaration of Helsinki, and the institutional review board (No. 4-2021-0416) approved the study.

## 3. Results

The final study population included 3,920,558 births, of which 275,009 (7.0%) were preterm (Figure 1). A slight male predominance was observed (51.3%). The proportion of preterm births was significantly higher during the NPI period (8.48%) compared to that in the pre-NPI period (6.92%) (*P *< 0.001). Additionally, the proportion of low-birth-weight neonates, with birth weight under 2,500 g, especially in the subcategory of 1,500 g to less than 2,500 g (5.09% vs. 5.98%, respectively, *P *< 0.001), was higher in the NPI period compared to the pre-NPI period. Detailed demographic features of the study populations for both periods are shown in Table 1.

**Table 1:** Clinical characteristics of study population in the pre-NPI and NPI periods.

		**Pre-NPI (January 2011–January 2020)**		**NPI (February 2020–December 2020)**		
	**Total**	***n***	**%**	***n***	**%**	***P* value**
**All births**	3,920,558	3,676,242		244,316		
**Sex**						
Male	2,012,375	1,887,286	51.34	125,089	51.20	0.19
**Gestational age**						
Preterm	275,009	254,287	6.92	20,722	8.48	< 0.001
Extremely preterm	10,134	9,419	0.26	715	0.29	0.0006
Very preterm	21,175	19,778	0.54	1,397	0.57	0.03
Moderate to late preterm	243,700	225,090	6.12	18,610	7.62	<0.001
37 weeks to <42 weeks, term	3,639,004	3,415,670	92.91	223,334	91.41	
≥ 42 weeks, post term	6,545	6,285	0.17	260	0.11	
**Birth weight**						
ELBW^1^	10,059	9,348	0.25	711	0.29	<0.001
VLBW^2^(excluding ELBW)	16,686	15,509	0.42	1,177	0.48	
LBW^3^(excluding VLBW and ELBW)	201,816	187,200	5.09	14,616	5.98	
2,500 g to <4,200 g	3,649,001	3,423,340	93.12	225,661	92.36	
≥ 4,200 g, macrosomia	42,996	40,845	1.11	2,151	0.88	
**Condition by birth weight and/or length**	3,774,408	3,541,932		232,476		
Small for gestational age (SGA)	232,569	219,985	6.21	12,584	5.41	<0.001
Large for gestational age (LGA)	371,336	348,236	9.83	23,100	9.94	0.10
**Type of birth**						
Singleton	3,774,618	3,542,128	96.35	232,490	95.16	<0.001
Multiple	145,414	133,602	3.63	11,812	4.83	
Missing	526	512	0.01	14	0.01	
**Birth order**						
1st	3,846,731	3,608,373	98.15	238,358	97.56	<0.001
2nd	72,048	66,204	1.80	5,844	2.39	
≥ 3rd	1,466	1,366	0.04	100	0.04	
Missing	313	299	0.01	14	0.01	
**Maternal age**						
<20 y	19,983	19,154	0.52	829	0.34	<0.001
20–24 y	181,277	172,705	4.70	8,572	3.51	
25–29 y	888,516	843,262	22.94	45,254	18.52	
30–34 y	1,535,255	1,449,809	39.44	85,446	34.97	
35–39 y	1,172,365	1,080,647	29.40	91,718	37.54	
≥ 42 y	122,643	110,152	3.00	12,491	5.11	
Missing	519	513	0.01	6	0.00	
**Maternal education**						
≤ Middle	71,238	67,468	1.84	3,770	1.54	<0.001
High	890,356	847,589	23.06	42,767	17.50	
College	2,926,851	2,734,939	74.39	191,912	78.55	
Missing	32,113	26,246	0.71	5,867	2.40	
**Season of birth**						
Spring (Mar-May)	1,014,810	944,907	25.70	69,903	28.61	<0.001
Summer (Jun-Aug)	962,406	895,263	24.35	65,117	26.65	
Autumn (Sep-Nov)	952,770	887,653	24.15	65,117	26.65	
Winter (Dec-Feb)	990,572	948,419	25.80	42,153	17.25	
**Delivery location, *N* (%)**						
Seoul, Gyeonggi-do	732,898	690,516	18.78	42,382	17.35	<0.001
Urban areas	991,065	930,897	25.32	60,168	24.63	
Other (rural area)	2,196,595	2,054,829	55.89	141,766	58.03	

^1^ ELBW, extremely low birth weight: 220 g to <1,000 g.

^2^ VLBW, very low birth weight: 1,000 g to <1,500 g.

^3^ LBW, low birth weight: 1,500 g to <2,500 g.

### 3.1. Preterm birth rate trends in the pre-NPI and NPI periods

**Figure 2:**
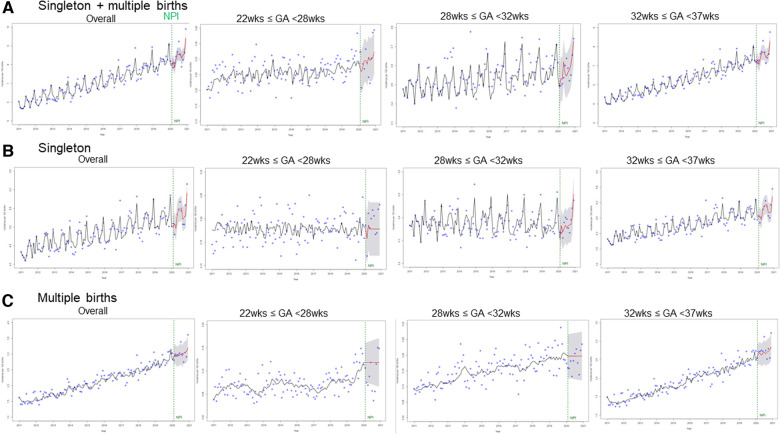
The observed values and expected monthly trend of the preterm birth rate (per 100 births) in the pre-NPI and NPI periods. (A) All births including singleton and multiple birhts, (B) Singleton births, (C) Multiple births The blue dots are observed values. The vertical green line denotes the period of NPI implementation. The black and red lines are trends for before and after NPI implementation, respectively. x-axis: incidence per 100 births, y-axis: year 2011–2021. NPI, non-pharmaceutical interventions.

The observed and expected preterm birth rates were plotted in Figure 2, by either birth type or GA subgroup. Overall, the preterm birth rates, including both singleton and multiple births, showed an increasing trend in all GA subgroups. (Figure 2, Supplementary Figure S1) However, based on the ARIMA model, NPI implementation was not associated with an immediate or further change in the preterm birth rate. For all singleton and multiple births, most observed values were within the 95% PI of the expected values, with an increasing trend (Figure 2A), except for those in July (observed 9.24, expected 8.54, [95% PI 8.13–8.96], percent difference 7.81%), September (observed 7.89, expected 8.35, [95% PI 7.93–8.76], percent difference −5.66%), and December (observed 9.90, expected 9.40, [95% PI 8.98–9.82], percent difference 5.2%) (Supplementary Table S1A). When preterm singleton births were separately analyzed, all the observed values were within the 95% PI (Supplementary Table S1B). For multiple births alone, most observed values were within the 95% PI, except for those in July (observed 3.39, expected 3.01 [2.68–3.35], percent difference 11.69%) and December (observed 3.61, expected 3.20 [95% PI 2.84–3.57], percent difference 11.94%) (Supplementary Table S1C).

### 3.2. Preterm birth rate and odds ratio of preterm births in the pre-NPI and NPI periods

Preterm births (including both singletons and multiple births) increased in the NPI period when compared to the pre-NPI period (odds ratio (OR) = 1.25, 95% CI, 1.23–1.27, *P *< 0.001) and a similar trend was observed in the multivariate analysis (OR = 1.18, 95% CI, 16–1.20, *P *< 0.001). (Table 2) When preterm births were separated into singleton and multiple birth groups and analyzed according to GA, both types of births showed similar trends with regards to preterm birth rates.

**Table 2:** Comparison of preterm births during the pre-NPI and NPI periods.

	**Pre-NPI (2011.01–2020.01) *n* (%)**	**NPI (2020.02–2020.12) *n* (%)**	**Unadjusted OR**^4^ **(95% CI)**	***P* value**	**Adjusted OR (95% CI)** ^†^	***P* value**
	3,676,242	244,316				
**Preterm birth by birth type**						
Singleton & preterm	174,959 (4.76)	13,006 (5.32)	1.13 (1.10, 1.15)	<0.001	1.11 (1.09, 1.13)	<0.001
Multiple & preterm	79,095 (2.15)	7,710 (3.16)	1.48 (1.45 1.52)	<0.001	1.39 (1.35, 1.43)	<0.001
Total & preterm	254,287 (6.92)	20,722 (8.48)	1.25 (1.23, 1.27)	<0.001	1.18 (1.16, 1.20)	<0.001
**Preterm birth by gestational age**						
Singleton						
Extremely preterm	6,496 (0.18)	466 (0.19)	1.08 (0.98, 1.19)	0.1107	0.99 (0.90, 1.08)	0.79
Very preterm	13,684 (0.37)	915 (0.37)	1.01 (0.94, 1.08)	0.8547	0.97 (0.91, 1.04)	0.40
Moderate to late preterm	154,779 (4.21)	11,625 (4.76)	1.14 (1.11, 1.16)	<0.001	1.13 (1.01, 1.15)	<0.001
Multiple						
Extremely preterm	2,781 (0.08)	247 (0.1)	1.34 (1.17, 1.52)	<0.001	1.11 (0.98, 1.27)	0.11
Very preterm	6,055 (0.16)	480 (0.2)	1.19 (1.09, 1.31)	0.0002	1.01 (0.92, 1.11)	0.81
Moderate to late preterm	70,259 (1.91)	6,983 (2.86)	1.51 (1.47, 1.55)	<0.001	1.40 (1.36, 1.44)	< 0.001

^4^ OR, odds ratio.

^†^ CI, confidence interval.

### 3.3. Global preterm birth rates during the pandemic

We summarized and compared the results of previous international studies on whether there was a change in the preterm birth rate due to NPI implementation (Table 3). Overall, there were nine international studies, and their defined lockdown periods ranged from 1 month to 12 months (19–22). A heterogeneity in outcomes was noted; however, the majority reported a decline in preterm birth rates in either the extremely preterm or late preterm groups (20, 23–27). There was also conflicting literature, that found no change in preterm births before and after NPI implementation in Spain, Sweden, and the United Kingdom (28–30). The results and design of these studies are described in Table 3.

**Table 3:** Global comparison of preterm birth rates during coronavirus disease 2019 lockdown.

	Study	Country	Study population	Sample size of exposed cohort (preterm births during lockdown)	Total sample size	Comparison period (pandemic group/control group)	Primary outcome and result
1	Been et al. 2020	Netherlands	National	56,720	1,599,547	March 9—July 16, 2020 (4 months)/Oct 9, 2010-March 8, 2020 (10 years)	Late preterm birth post mitigation measures decreased (24)
2	Hedermann, et al. 2021	Denmark	National	5,162	31,180	March 12-Apr 14, 2020 (1 month)/Annual matched (2015–19)	Preterm birth before 28 weeks’ gestation decreased (90%) (21)
3	Caniglia et al. 2020	Botswana	National	10,751	68,448	April 3 -Jul 20, 2020 (3 months)/Annual matched 2017–19	Preterm birth before 37 weeks gestation post mitigation measures decreased (39)
4	Kim et al. 2021	Korea	Single center	246	3,011	March 22 2020-Oct 31, 2020 (7 months)/Annual matched 2011–2019	Preterm birth rate decreased from 14.5% to 8.5% (38)
5	Bian et al. 2021	China	National	24,312	164,107	January 23 2020-June 2020/July 2020-Dec 2020 (mitigation relaxed)	Late preterm birth decreased (OR 0.84) (23)
6	Philip et al. 2020	Ireland	National	1,381	93,018	January -April 2020 (4 months)/Annual matched 2001–2020	73% reduction of very low birth weight infants (20)
7	Matheson et al. 2021	Australia	3 multi-centers	2,427	27,760	July 2020-Sep 2020 (3 months)/Jan 2018-May 2020	Preterm birth rate decreased (before 28 weeks, 0.4% vs. 0.8%), before 34 weeks (2.6% vs. 3.6%), and before 37 weeks, 8.3% vs. 10.1%) (40)
8	Oakley et al. 2022	Norway, Denmark	Multi-national	85,098	1,519,521	March 12-Jun 12, 2020 (4 months)/March 2014–2019	No decline in preterm births in all three countries (8)
9	De Curtis et al. 2020	Italy	Single center	419	16,808	March-May 2020 (3 months)/Annual matched 2019	Decline in late preterm birth (5.93% vs. 4.62%) (8, 25)
10	Pasternak et al. 2021	Sweden	Single center	801	108,923	Apr 1, 2020- May 31, 2020 (2 months)/Annual matched 2015–2019	No association (28)
11	Khalil et al. 2020	UK	Single center	189	3,462	October 1, 2019-Jan 31, 2020 (4 months)/Feb 1, 2020-June 14, 2020	Non-significant increase in preterm births for gestations less than 34 weeks and 37 weeks (30)
12	Meyer et al. 2021	Israel	Single center	226	31,428	March 20 to June 27, 2020 (4 months)/Annual matched 2011–2019	Preterm birth rate at less than 34 weeks of gestation was significantly lower in the lockdown period compared to matched 2011–2019 (OR = 0.45 [95% CI, 0.30,0.68] and OR = 0.60 [95% CI, 0.41,0.85], respectively (26)
13	Arnaez et al. 2020	Spain	National	4,528	70,024	March 15, 2020-June 21, 2020 (1 month)/Jan 2015-June 2020	No change during lockdown (OR 1.38, *p* = 0.438) (29)
14	Handley et al. 2020	USA	Multi-center	283	8,867	March-June 2020 (4 months)/Annual matched 2018–2020	No significant change in preterm or stillbirth rates during the pandemic (10.5% vs. 9.5%) (41)
15	KC et al. 2020	Nepal	National	3,467	10,543	March 21, 2020-May 3–2020 (2 months)/Jan 1-March 20, 2020	Preterm birth rate increased by 16.5% (aRR 1.3) during the lockdown (41, 42)
16	Kirchengast S et al.	Austria	Single center	52	29,753	March -July 2020 (5 months)/Jan-Feb 2020, annual matched 2005–2019	Rate of very preterm birth during the lockdown months was markedly lower than that pre-lockdown. No significant decrease in late preterm birth rate was observed (OR 1.01, CI 0.97–1.05) (43)
17	Huseynova et al. 2021	Saudi Arabia	Single center	266	7,226	March 1-June 30, 2020 (4 months)/2017–2019, annual matched 2020	23% reduction in the birth rate of extremely preterm and moderate/late preterm infants during the lockdown (26, 27)
18	Fresson et al. 2022	France	National	4,799	96,076	March 17- May 10, 2020 (2 months)/Jan 1, 2016-July 31 2020	Decline in late preterm birth was observed (OR 0.92) (44)
19	Shah et al., 2021	Canada	Regional	194,025	2,465,387	January-December 2020 (12 months)/July 2002—Dec 2020	No change in preterm birth rate (45)

## 4. Discussion

In this study, we aimed to establish whether a correlation exists between preterm birth rates and the NPI period in Korea in 2020. Our findings showed that the preterm birth rate was increasing in the pre-NPI period, and this continued to increase after the implementation of such measures, suggesting an unclear impact of NPI implementation on the preterm birth rate.

Preterm births have been increasing in most developed countries, including South Korea. The percentage of preterm births in the United States increased from 9% in 1981 to 10.5% in 2021 (31, 32). Similar trends have also been observed in East Asian countries such as Taiwan and South Korea (33). Increased accessibility to medical services, increased number of survivors of chronic diseases which were previously fatal, and the development of assisted reproductive technologies have contributed to an overall increase in high-risk pregnancies such as twins or those of advanced maternal age (34).

The etiology of preterm delivery can be broadly categorized into three main groups: (1) spontaneous preterm labor; (2) maternal or fetal infections; and (3) premature preterm rupture of the membranes (9). Etiologies for preterm birth are generally unclear; however, environmental factors, such as viral infection and smoking exposure, are important risk factors for both ruptured and intact membrane preterm labor (35). NPI could change maternal environmental and habitual exposures, leading to a possible change in preterm birth rates. Bian et al. suggested that changes in the overall behavior of pregnant women during the pandemic might also have contributed to the above, as one-third of preterm births are iatrogenic (23, 36).

In contrast to our findings, many international data-based studies supported a negative association between NPI and preterm birth that were investigated up to the first quarter of 2020. In the United States, Gemmill et al. reported a decline in preterm birth rate during the pandemic, especially during the early and late 2020 (37). Oakley et al. observed a decrease in preterm births after NPI initiation until March 2020 in Norway, Sweden, and Denmark (8). Hedermann et al. also noted this phenomenon among the Danish population born before February 2020 (21). Bian et al. investigated births up to December 2020 in a single center study in Shanghai, China, and found a significant decrease in preterm birth rates (23). Kim et al., in a Korean single center study, reported a decrease in preterm labor after the NPI period (38). Serial works of published data that followed support similar associations of decreased preterm birth rates with the COVID-19 lockdown (Table 3).

### 4.1. Limitations

Owing to the lack of confirmed etiologies for preterm births, the decline in preterm births during pandemic lockdowns could simply be a coincidence. On the other hand, further investigating this potential association could lead to more effective measures to prevent preterm births. Cohort data from other countries with lockdown periods during the pandemic showed a potential decrease in preterm births during the pandemic; however, they only investigated a short period of time after the NPI, mostly the first three months of 2020. This contrasts with our study, with data collection over a large period, in which we found no significant relationships between NPI implementation and preterm birth. Another limitation to our study is the lack of individual patient data in our database. For instance, the database does not include information on the cause or type of preterm birth, such as an underlying maternal or fetal condition (“indicated preterm birth”) or preterm rupture of membranes or cervical dilatation (“spontaneous preterm birth”). Consequently, it is difficult to correlate NPI with a specific type of preterm birth. However, because NPI could affect the maternal or fetal environment in multifaceted ways, differentiating the type of birth in the analysis may not have necessarily provided additional helpful information. However, to improve the quality of the database, further collection of National Health Insurance Data and the Korean Developmental Survey Data is required in the future. As a result of these limitations, we cannot definitively conclude that a causal relationship between COVID-19 mitigation measures and preterm birth rate exists.

## 5. Conclusions

In conclusion, we were unable to establish a significant relationship between the NPI and pre-NPI preterm birth rates in South Korea. The overall South Korean preterm birth rate continues to increase, and this contributes to the country's disease burden. Future research should ideally aim to investigate whether there are more significant variables that could affect preterm birth rates, as well as how clinicians can reduce these factors and ultimately decrease the rate of preterm births.

## Data Availability

Birth registry data from the Korean Statistical Information Service (KOSIS) can be accessed from the KOSIS website. The raw data from the KOSIS is available at https://kosis.kr.
