# Supplementary material for: The impact of non-pharmaceutical interventions on premature births during the COVID-19 pandemic: a nationwide observational study in Korea
Source: Front Pediatr. 2023 Jun 27;11:1140556. doi: 10.3389/fped.2023.1140556 (PMC10333519; doi:10.3389/fped.2023.1140556)
Supplement: Supplementary file 1 [file Datasheet1.pdf]

frontiers

Supplementary Material

The Impact of Non-Pharmaceutical Interventions on Premature Births during the COVID-19 Pandemic: A Nationwide Observational Study in Korea

Ji Young Lee[1†], Joonsik Park[2†], Myeongjee Lee[3†], Minkyung Han[3], Inkyung Jung[4], Sung Min Lim[1], Jee Yeon Baek[1], Ji-Man Kang[1,5], Min Soo Park[1,2,6,7]*, Jong Gyun Ahn[1,5]*,

*** Correspondence:** Min Soo Park, MD, PhD: minspark@yuhs.ac

Jong Gyun Ahn, MD, PhD: JGAHN@yuhs.ac

1 Supplementary Figures and Tables

1.1 Supplementary Figures

Segmented Equation 1

$$Y_t = \beta_0 + \beta_1 * time_t + \beta_2 * transition\_NPI_t + \beta_3 * transition\_NPI_t + v_t$$

$$v_t = -\gamma_1\ v_{t-1} - \gamma_2\ v_{t-2} - \ldots - \gamma_k\ v_{t-k} + \varepsilon_t,$$

where v t autogressive error model of order k,

β0 baseline level of the incidence at time t=0, January 2008

β1 average monthly change in the incidence before the transition to NPI

β2 the immediate effect of the transition, i.e., the change in level (drop or jump) of the incidence immediately after the transition

β3 the change in the slope (increase or decrease) of the incidence after the transition to NPI, compared to the slope for the segment before the transition

β1+ β3 the postintervention slope of the segment after the transition, i.e., the average percentage increase in the incidence from 1 month to the next after the transtion to the NPI coding.

Reference: Wagner AK, Soumerai SB, Zhang F, Ross-Degnan D. Segmented regression analysis of interrupted time series studies in medication use research. *J Clin Pharm Ther.* 2002;27(4):299-309, https://doi.org/10.1046/j.1365-2710.2002.00430.x.

eFigure 1. Annual incidence of preterm birth rates before and during the COVID-19 non-pharmaceutical intervention period using segmented regression analysis.

A. Single and multiple births

1) Preterm (overall)

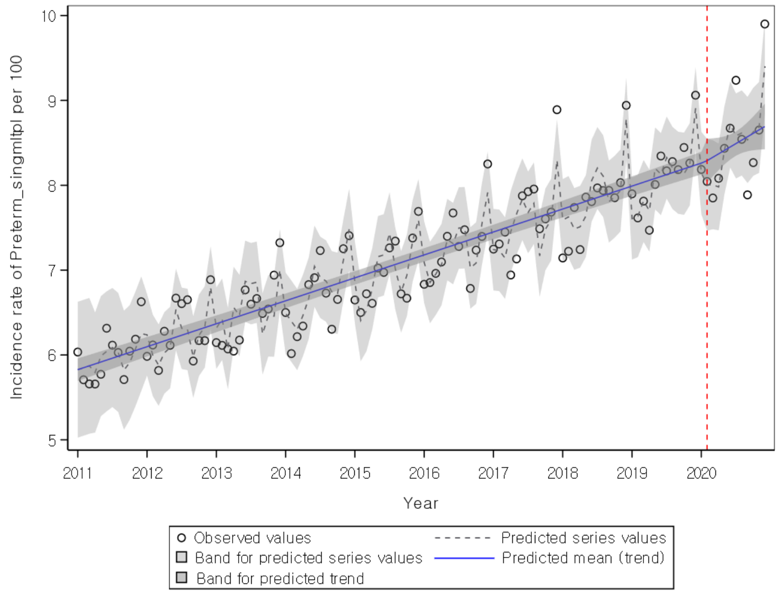

2) Extremely preterm (22weeks <=GA[1] <28 weeks)

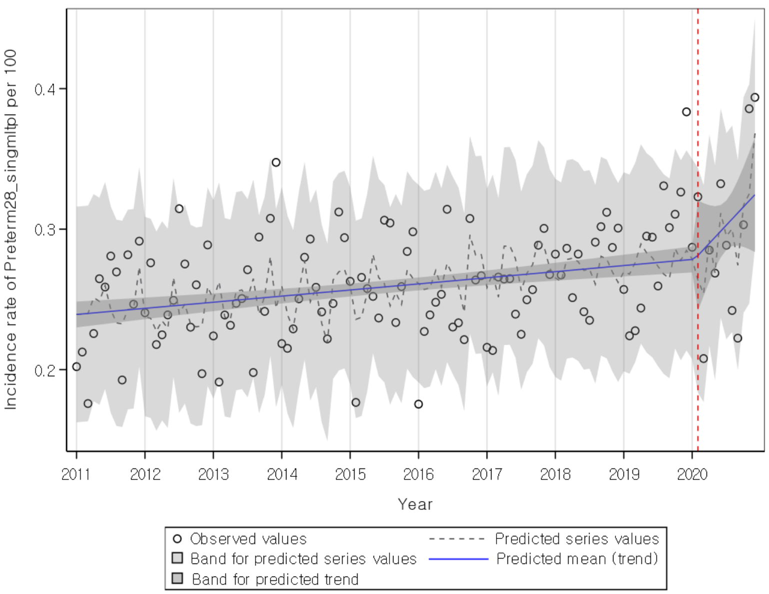

* Red dotted line marks NPI period

[1] GA, gestational age

3) Very preterm (28weeks <=GA <32 weeks)

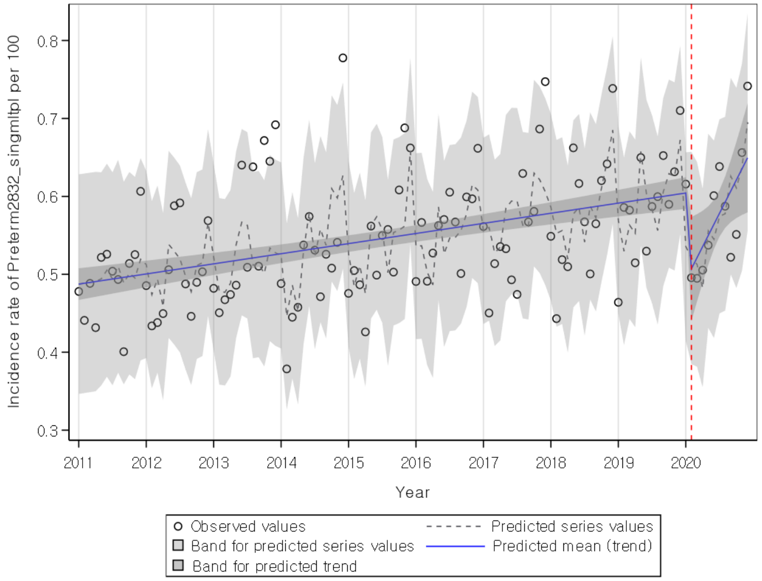

4) Moderate to late preterm (32weeks <=GA <37weeks)

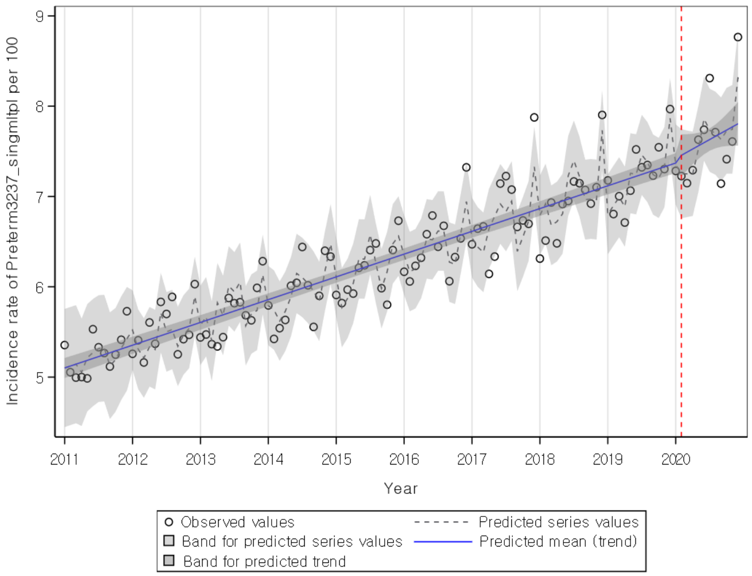

B. Single births

1) Preterm (overall)

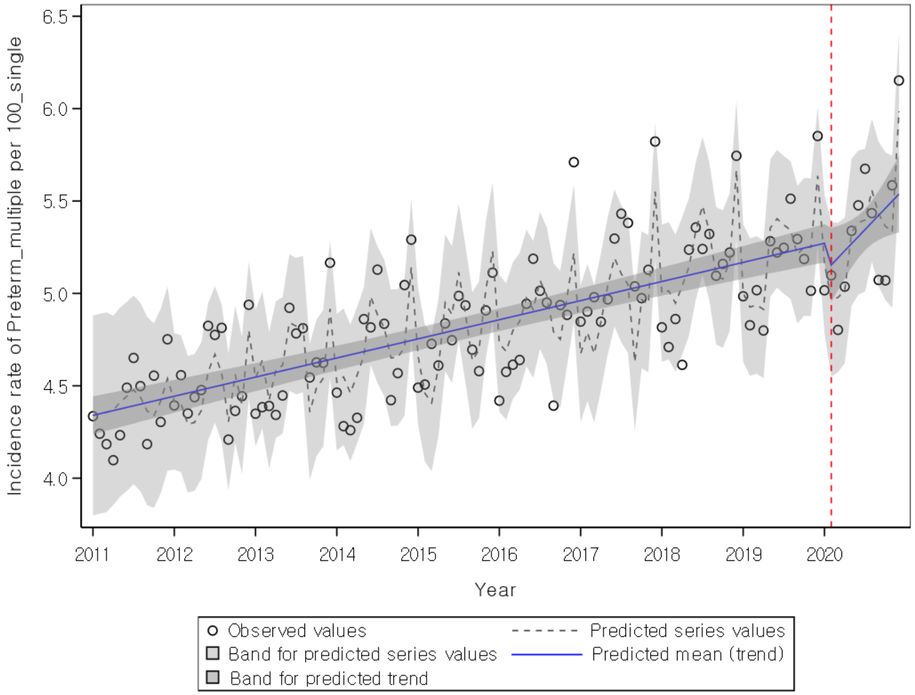

2) Extremely preterm (22weeks <=GA <28 weeks)

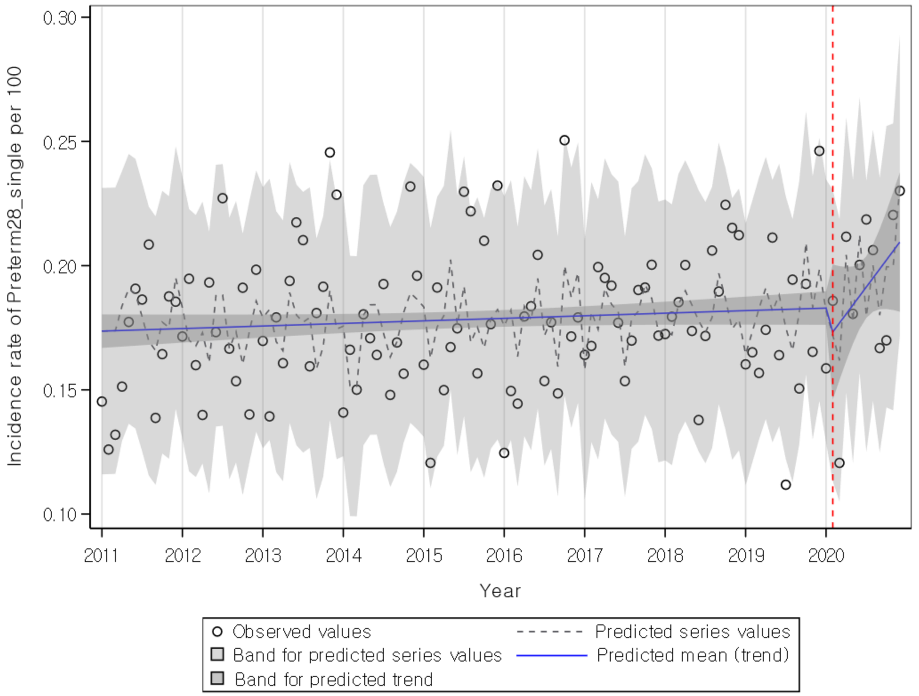

[1] GA, gestational age

3)Very preterm (28weeks <=GA <32 weeks)

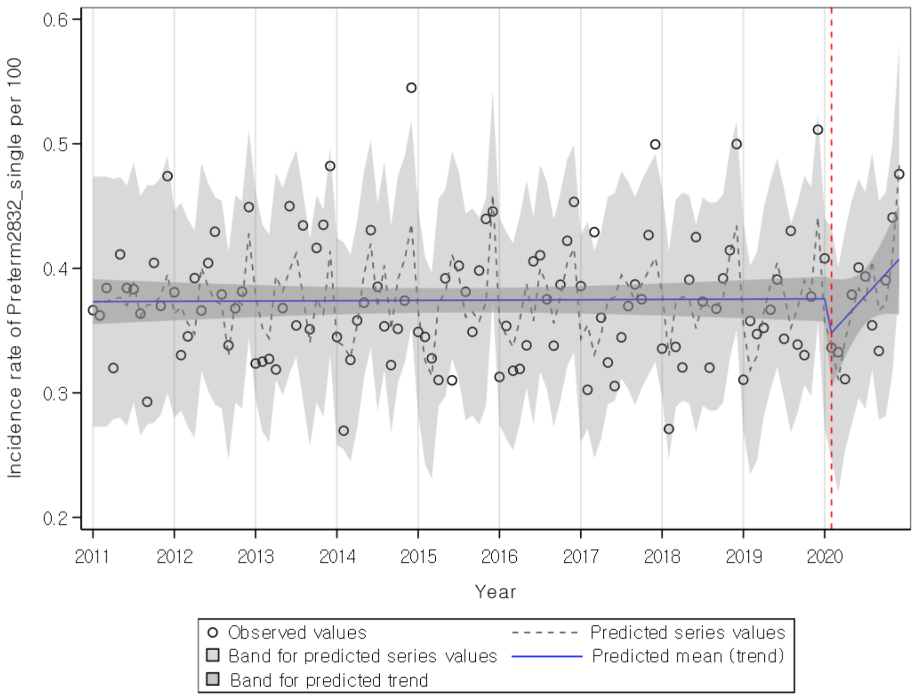

4) Moderate to late preterm (32weeks <=GA <37weeks)

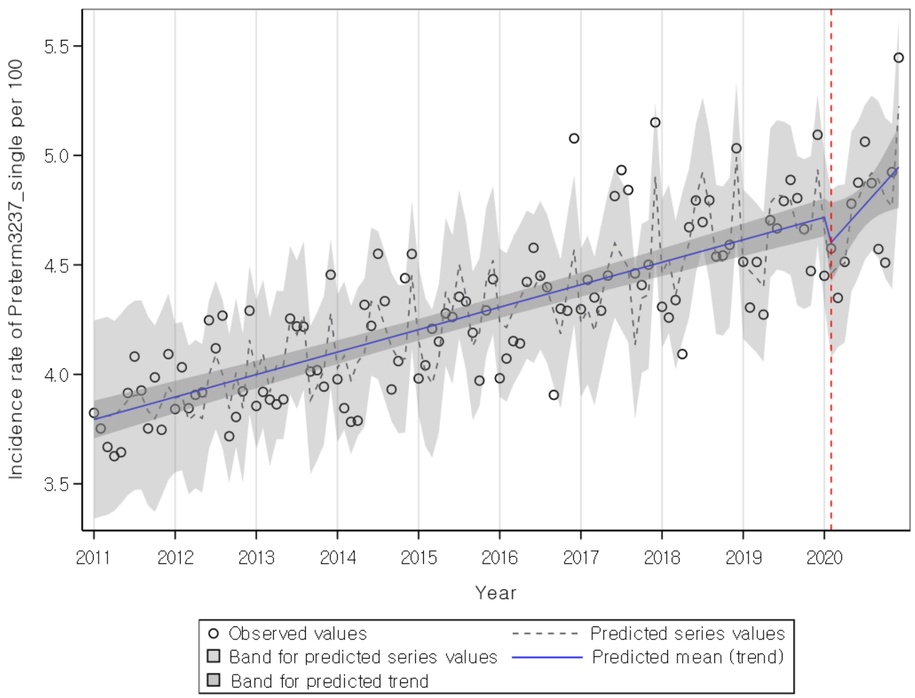

C. Multiple births

1) Preterm (overall)

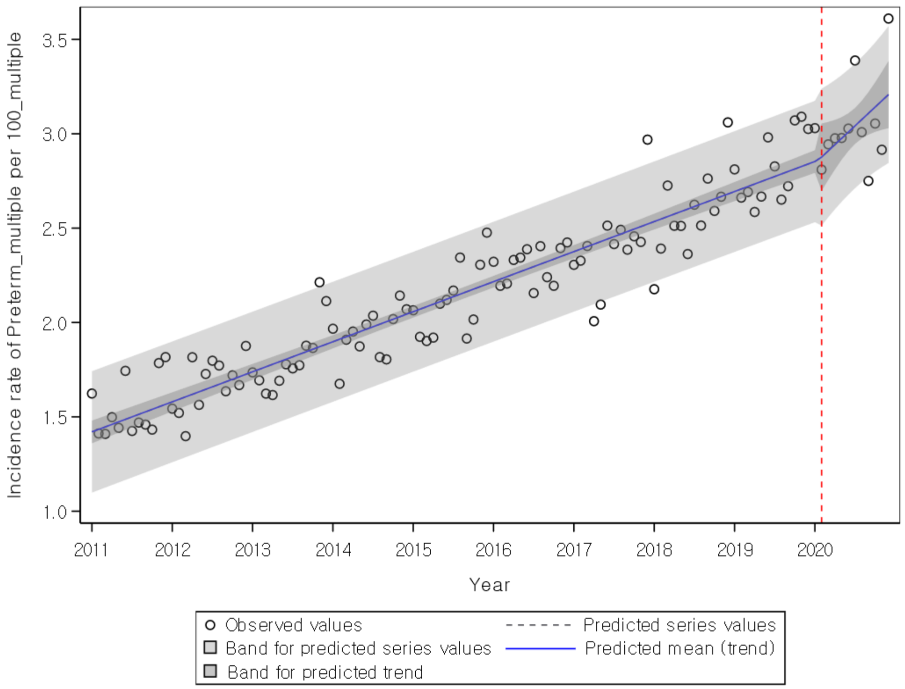

2) Extremely preterm (22weeks <=GA <28 weeks)

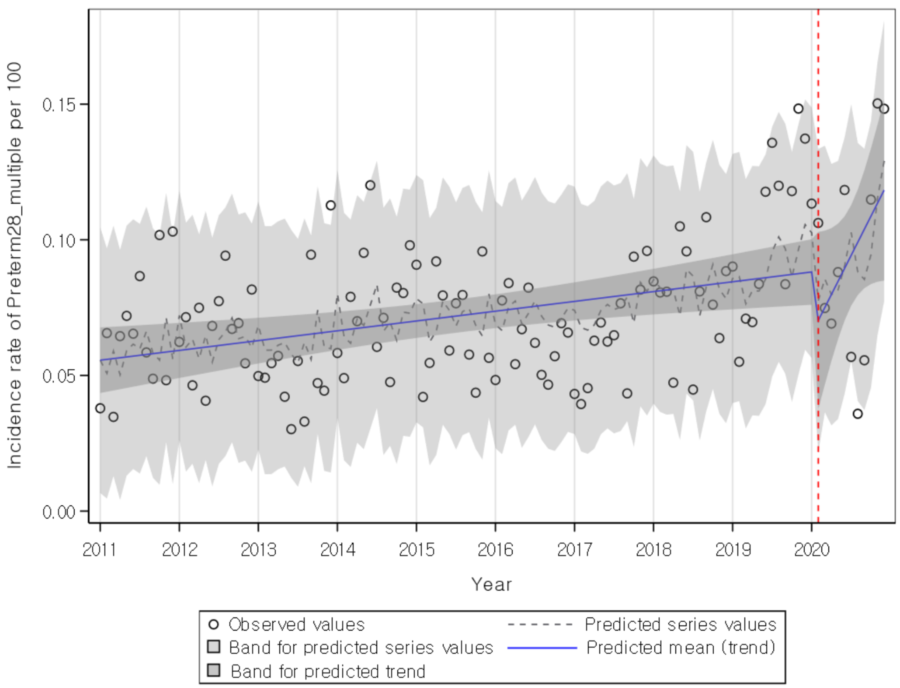

[1] GA, gestational age

3)Very preterm (28weeks <=GA <32 weeks)

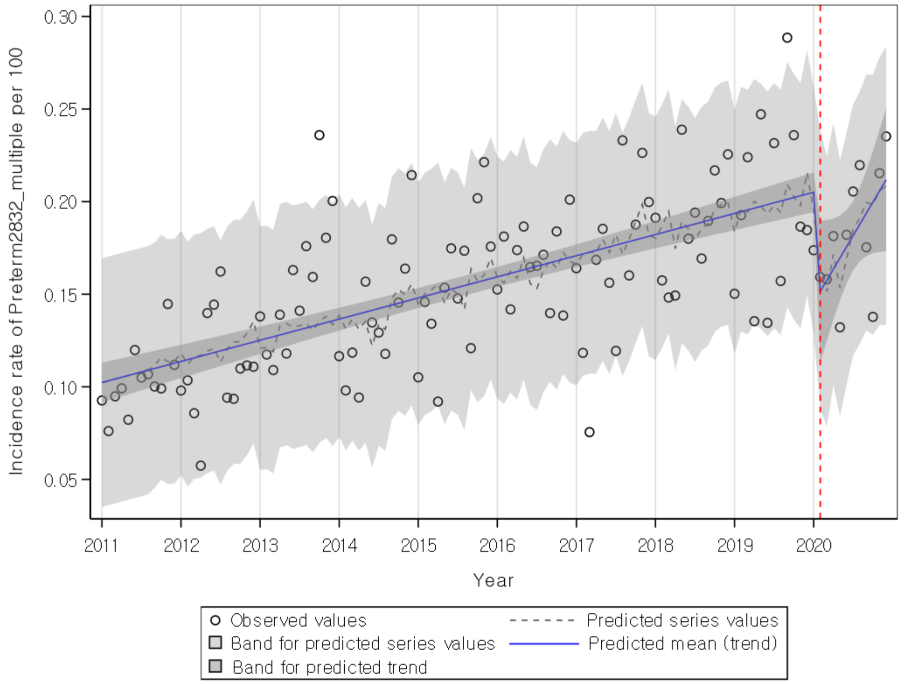

4) Moderate to late preterm (32weeks <=GA <37weeks)

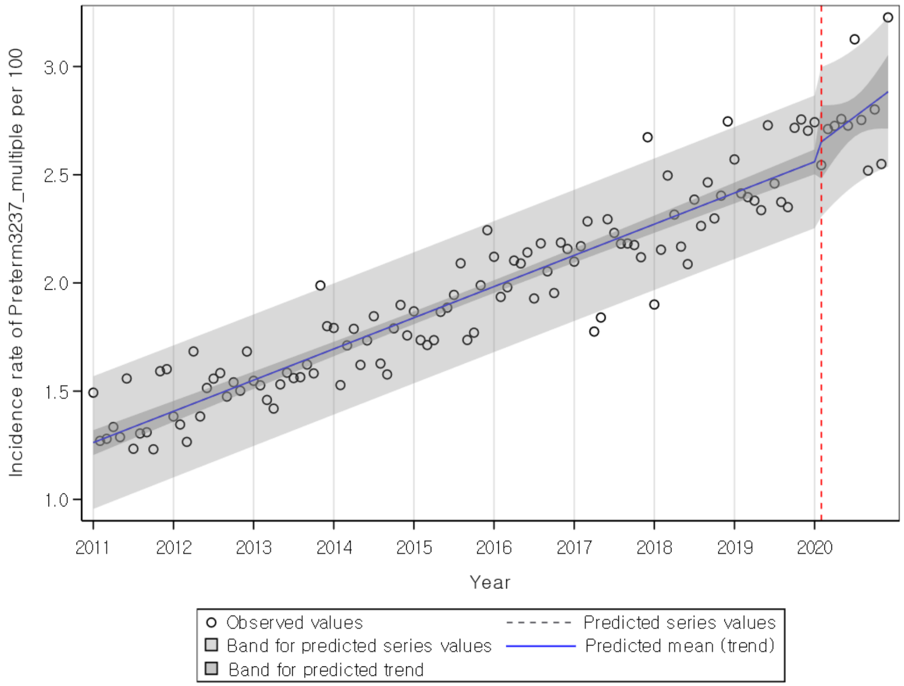

1.2 Supplementary tables

eTable 1. ARIMA models of preterm birth incidence per 100 live births after NPI implementation (numerical values for Figure 2)

A) Singleton and multiple births

[1] GA, gestational age

Overall					22wk<=GA<28wks				28wk<=GA<32wks				32wk<=GA<37wks			
	Observed	Expected	Expected 95% PI	percent difference	Observed	Expected	Expected 95% PI	percent difference	Observed	Expected	Expected 95% PI	percent difference	Observed	Expected	Expected 95% PI	percent difference
Februray	8.04	7.91	7.50-8.33	1.63	0.32	0.27	0.20-0.34	18.08	0.50	0.49	0.37-0.60	2.02	7.23	7.13	6.73-7.52	1.36
March	7.85	8.15	7.74-8.57	-3.8	0.21	0.26	0.19-0.33	-22.16	0.49	0.53	0.42-0.65	-7.32	7.15	7.32	6.92-7.71	-2.35
April	8.08	7.85	7.44-8.27	2.88	0.29	0.28	0.21-0.34	3.43	0.51	0.53	0.41-0.64	-4.62	7.29	7.06	6.66-7.45	3.29
May	8.44	8.30	7.88-8.72	1.62	0.27	0.29	0.22-0.36	-9.06	0.54	0.62	0.51-0.74	-14.39	7.63	7.36	6.97-7.76	3.56
June	8.67	8.58	8.16-9.00	1.07	0.33	0.30	0.23-0.37	11.53	0.60	0.56	0.44-0.67	7.52	7.74	7.71	7.31-8.10	0.43
July	9.24	8.54	8.13-8.96	7.81	0.29	0.28	0.21-0.35	1.73	0.64	0.55	0.43-0.66	15.53	8.31	7.68	7.29-8.08	7.83
August	8.54	8.59	8.17-9.01	-0.53	0.24	0.31	0.24-0.38	-23.44	0.59	0.57	0.45-0.68	3.85	7.71	7.68	7.28-8.08	0.42
September	7.89	8.35	7.93-8.76	-5.66	0.22	0.30	0.23-0.37	-28.49	0.52	0.58	0.46-0.69	-10.29	7.14	7.43	7.03-7.82	-3.89
October	8.27	8.50	8.08-8.92	-2.81	0.30	0.30	0.23-0.37	1.19	0.55	0.60	0.49-0.72	-9.22	7.41	7.55	7.15-7.94	-1.77
November	8.65	8.58	8.16-9.00	0.85	0.39	0.30	0.23-0.38	23.45	0.66	0.66	0.54-0.77	0.03	7.61	7.59	7.20 -7.99	0.2
December	9.90	9.40	8.98-9.82	5.2	0.39	0.32	0.25-0.39	19.63	0.74	0.73	0.61-0.85	1.62	8.77	8.30	7.90-8.70	5.41

B) Singleton births

	Overall				22wk<=GA<28wks				28wk<=GA<32wks				32wk<=GA<37wks			
	Observed	Expected	Expected 95% PI	percent difference	Observed	Expected	Expected 95% PI	percent difference	Observed	Expected	Expected 95% PI	percent difference	Observed	Expected	Expected 95% PI	percent difference
Februray	5.10	4.99	4.67-5.30	2.22	0.19	0.18	0.13 - 0.23	3.16	0.34	0.30	0.23-0.38	10.47	4.58	4.50	4.21-4.80	1.59
March	4.80	5.09	4.78-5.41	-5.86	0.12	0.16	0.10 - 0.21	-26.62	0.33	0.37	0.29-0.44	-9.4	4.35	4.56	4.26-4.86	-4.79
April	5.04	4.96	4.64-5.27	1.62	0.21	0.19	0.13 - 0.24	12.58	0.31	0.34	0.26-0.41	-7.74	4.51	4.44	4.14-4.74	1.62
May	5.34	5.33	5.01-5.65	0.12	0.18	0.18	0.12 - 0.24	1.32	0.38	0.35	0.28-0.43	6.8	4.78	4.76	4.46-5.07	0.33
June	5.48	5.46	5.13-5.78	0.37	0.20	0.18	0.12 - 0.24	11.66	0.40	0.37	0.29-0.44	8.8	4.88	4.89	4.58-5.19	-0.25
July	5.67	5.47	5.15-5.80	3.63	0.22	0.18	0.12 - 0.24	20.34	0.39	0.35	0.28-0.43	10.77	5.06	4.93	4.63-5.24	2.59
August	5.43	5.52	5.19 - 5.84	-1.54	0.21	0.18	0.12 - 0.24	14.57	0.35	0.37	0.29-0.44	-3.3	4.87	4.94	4.63-5.25	-1.37
September	5.07	5.21	4.88 - 5.54	-2.63	0.17	0.18	0.12 - 0.24	-6.62	0.33	0.37	0.29-0.44	-9.76	4.57	4.68	4.36-4.99	-2.24
October	5.07	5.27	4.94 - 5.60	-3.79	0.17	0.18	0.12 - 0.24	-4.77	0.39	0.37	0.29-0.45	5.37	4.51	4.68	4.37-5.00	-3.74
November	5.59	5.31	4.97 - 5.64	5.12	0.22	0.18	0.12 - 0.24	21.15	0.44	0.41	0.33-0.49	7.22	4.92	4.70	4.38-5.02	4.65

[1] GA, gestational age

December	6.15	5.93	5.59 - 6.26	3.75	0.23	0.18	0.12 - 0.24	25.42	0.48	0.50	0.43-0.58	-5.49	5.45	5.20	4.88-5.52	4.57

C) Multiple Births

	Overall				22wk<=GA<28wks				28wk<=GA<32wks				32wk<=GA<37wks			
	Observed	Expected	Expected 95% PI	percent difference	Observed	Expected	Expected 95% PI	percent difference	Observed	Expected	Expected 95% PI	percent difference	Observed	Expected	Expected 95% PI	Percent difference
Februray	2.81	2.90	2.60-3.20	-3.16	0.11	0.12	0.08-0.16	-10.92	0.16	0.20	0.13-0.27	-21.24	2.54	2.60	2.30-2.90	-2.13
March	2.94	3.02	2.71-3.33	-2.51	0.07	0.12	0.08-0.16	-44.98	0.16	0.20	0.13-0.27	-22.05	2.71	2.67	2.37-2.97	1.51
April	2.98	2.92	2.61-3.24	1.76	0.07	0.12	0.07-0.16	-52.48	0.18	0.20	0.13-0.27	-8.32	2.73	2.68	2.38-2.97	1.84
May	2.98	2.94	2.62-3.26	1.22	0.09	0.12	0.07-0.16	-29.25	0.13	0.20	0.12-.27	-39.48	2.76	2.63	2.32-2.93	4.83
June	3.03	2.96	2.63-3.29	2.26	0.12	0.12	0.07-0.16	0.07	0.18	0.20	0.12 - 0.27	-7.94	2.73	2.67	2.36-2.99	2.00
July	3.39	3.01	2.68-3.35	11.69	0.06	0.12	0.07-0.17	-70.18	0.21	0.20	0.12-0.27	4.13	3.13	2.71	2.39-3.03	14.29
August	3.01	2.94	2.60-3.28	2.35	0.04	0.12	0.07-0.17	-106.92	0.22	0.20	0.12-0.27	10.81	2.75	2.66	2.33-2.99	3.42
September	2.75	3.04	2.69	-9.94	0.06	0.12	0.07-0.17	-72.09	0.18	0.20	0.12-0.27	-11.7	2.52	2.71	2.37-3.05	-7.37

October	3.05	3.06	2.70	-0.06	0.11	0.12	0.07-0.17	-2.97	0.14	0.20	0.12-0.27	-35.45	2.80	2.73	2.38-3.08	2.62
November	2.92	3.09	2.73	-5.67	0.15	0.12	0.07-0.17	23.83	0.22	0.20	0.12-0.27	8.84	2.55	2.77	2.41-3.12	-8.11
December	3.61	3.20	2.84	11.94	0.15	0.12	0.07-0.17	22.53	0.24	0.20	0.12-0.27	17.62	3.23	2.85	2.49-3.21	12.37

[1] GA, gestational age
